# Type II Acute Macular Neuroretinopathy Secondary to Malaria

**DOI:** 10.1155/2024/1577127

**Published:** 2024-05-29

**Authors:** Alastair David Bezzina, Jeremy Spiteri Bailey, Isaac Bertuello

**Affiliations:** Ophthalmology Department Mater Dei General Teaching Hospital, Msida, Malta

## Abstract

To the best of our knowledge, we present the first case of type II acute macular neuroretinopathy (AMN) exhibiting in a patient suffering from malarial retinopathy concomitant with cerebral malaria acquired after travelling to West Africa without taking the necessary antimalarial prophylaxis. The patient complained of bilateral blurring of vision after being removed off sedation whilst at the intensive care unit. Subsequent examination revealed bilateral retinal haemorrhages, cotton-wool spots, and foveal pigmentary changes in keeping malarial retinopathy. Macular optical coherence tomography (OCT) revealed patchy hyperreflective changes at the level of the outer plexiform and outer nuclear layers (ONL) in keeping with the areas of deep capillary plexus flow void noted on OCT-angiography (OCT-A). This case report sheds more light on the extent of neurosensory retinal ischaemia in malarial retinopathy and showcases a new imaging biomarker which may be utilized in assessing and quantifying the functional deficit created by this disease.

## 1. Introduction

Type II AMN is an ischaemic event which occurs at the level of the deep capillary plexus [1]. Symptoms are generally limited to central and paracentral scotomata, and fundal examination may only reveal small hypopigmented wedge-shaped lesions within the macular area, given that there is no associated retinopathy [2]. AMN has been associated with multiple aetiologies including malarial retinopathy [3], but, to our knowledge, this is the first documented case of standalone type II AMN occurring in the context of the aforementioned disease.

## 2. Case Report

The patient in question is a 41-year-old Caucasian male who was brought by his wife to the emergency department in view of sudden onset confusion, incoherent speech, agitation, and generalized stiffness with fixed flexion of the upper limbs.

A collateral history was obtained from the wife which revealed that the patient began to feel unwell six days after returning home from overseas. Initially, the patient experienced fever, bouts of vomiting, and diarrhea associated with myalgias and arthralgias. He was seen by his general practitioner and given the preliminary diagnosis of traveler's diarrhea and prescribed azithromycin three days prior to admission to the emergency department in view of his persistent symptomatology. The patient showed no improvement and deteriorated rapidly prior on the same day of presentation to the emergency department.

He was not on any regular medications, and the only surgical history of note was a splenectomy performed due to previous trauma.

The patient in question was otherwise previously independent with no history of drug or alcohol abuse.

He had recently returned to Malta from a trip to West Africa where he travelled and stayed in Liberia, Ghana, and Guinea-Bissau. The recommended vaccinations had been administered prior to his travels; however, the patient was found to have abstained from the use of antimalarial prophylactic medications.

Initial examination revealed a GCS (Glasgow Coma Scale) of 12 which deteriorated to GCS 10 whilst at the emergency department. Pupils were equal and reactive to light. A blood pressure of 150/70 mmHg, pulse of 97 bpm, respiratory rate of 18 rpm, O2 saturation of 96% (on room air), and a temperature of 37.8°C were recorded on preliminary examination.

Initial investigations included venous blood gases which revealed a pH of 7.32, pCO2: 49.4 mmHg, pO2: 28.9 mmHg, lactate: 4.5 mmol/L, and glucose: 5.9 mmol/L.

Laboratory tests showed an elevated white cell count of 13.4 (4.30-11.40 × 10^9^/L), an elevated level of dysplastic neutrophils of 11.88 (2.10-7.20 × 10^9^/L) with toxic granulations, thrombocytopenia with a platelet count of 59 (146-302 × 10^9^/L), a red blood cell count of 0.08 (3.90-5.40 × 10^12^/L), a haematocrit level of 21% (35.6-46%) and a hemoglobin count of 6.6 (14.1-17.2 g/dL). Microscopic examination of the blood film revealed parasitic infiltration of the red blood cells (ring stages and Schizont forms observed). The C-reactive protein level was raised to 132.5 mg/dL. Liver function tests revealed a bilirubin level of 147.1 (0-21 micromol/L), raised serum alkaline phosphate 190 (40-129 U/L), raised serum gamma-glutamyl transferase 254 (8-61 U/L), and alanine transferase levels 83 (5-41 U/L). The renal profile was unremarkable. The malaria rapid diagnostic test resulted positive.

Initial imaging included a chest X-ray and CT brain both of which did not show any abnormalities.

Given the patient's poor GCS, he was admitted to the intensive care unit with suspected cerebral malaria. Lumbar puncture for cerebrospinal fluid analysis was not performed at this point due to concurrent thrombocytopenia. The patient was started on artesunate, ceftriaxone, dexamethasone, and acyclovir.

An electroencephalogram was performed by the caring neurologists which showed slow background cerebral activity and frequent triphasic sharp waves suggestive of generalized toxic/metabolic encephalopathy without any definite focal features.

Magnetic resonance imaging of the brain was also conducted which demonstrated bilateral and mostly symmetrical foci of intracranial restricted diffusion with corresponding apparent diffusion coefficient map abnormalities at the level of the thalami as well as the basal ganglia.

After 1 week in the intensive therapy unit, the patient was extubated after showing good progress and transferred to the ward two days later.

After the removal of sedation, the patient noted that he still had his contact lenses in situ and immediately started complaining of persistent “fogginess” in both visual fields even after removing his lenses and wearing his glasses for the following 2 days. This prompted the caring infectious diseases team to contact the ophthalmologist on-call for a review. On presentation to the ophthalmic clinic, the patient denied the presence of a dense central scotoma and/or metamorphopsia and any past ophthalmic history of note.

Initial examination revealed the following: right eye visual acuity (VA) of 6/9 (Snellen) and left eye VA of 6/9; no relative afferent pupillary defect was observed, and the patient managed to read all 13 Ishihara pseudoisochromatic plates with both eyes. Confrontational visual field testing was unremarkable. The anterior segment was unremarkable on slit-lamp biomicroscopy of note with quiet anterior chambers normal intraocular pressure readings and clear lens media.

**Figure 1:**
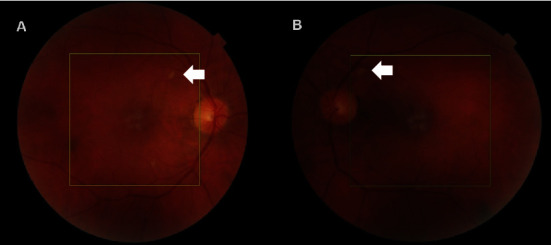
Fundal photography demonstrating cotton wool spots (arrow) in the (A) right eye and (B) left eye.

Posterior segment examinations revealed patchy hypopigmentary changes involving the posterior pole in both eyes with associated cotton wool spots in the left fundus (Figure 1).

**Figure 2:**
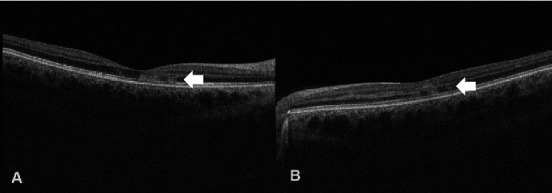
OCT B-scan demonstrating patchy hyperreflective changes at the level of the OPL and ONL (arrows) in the (A) right and (B) left eyes.

**Figure 3:**
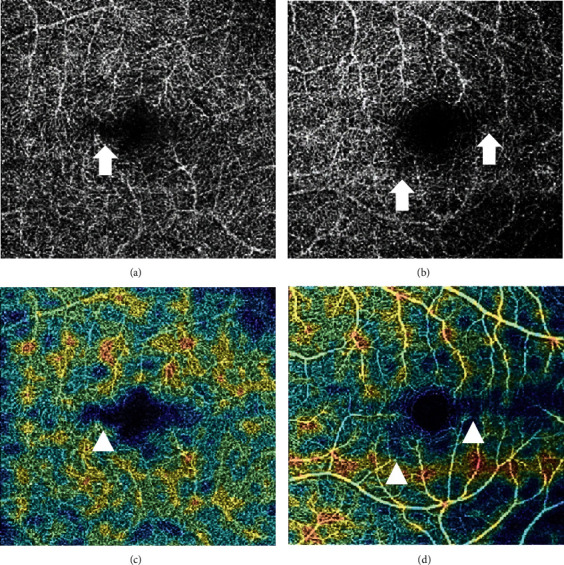
Deep capillary plexus flow voids in the perifoveal area of the (a, arrow) right eye and (b, arrow) left eye with enlargement of the foveal avascular area noted on the composite angiographic density map in the respective eyes ((c, arrowhead) right eye and (d, arrowhead) left eye).

OCT imaging of the macula revealed patchy hyperreflective bands within the OPL and ONL layers in both maculae (Figure 2) with corresponding flow voids on OCT-A noted at the level of the deep capillary plexus in both eyes (Figure 3).

A diagnosis of bilateral malarial retinopathy with associated type II AMN was made. The patient was advised that treatment for this condition, apart from tackling the cause of the retinopathy, was not available and that only conservative management and follow-up to monitor its progress and deal with any possible sequelae could be offered.

Unfortunately, the patient did not attend his follow-up appointments after being discharged from the hospital, and the current status of the retina could not be determined.

## 3. Discussion

Malaria is a zoonotic infection caused by protozoan species falling under the *Plasmodium* genus [4]. According to the 2023 World Health Organization malaria report, 249 million cases of malaria have been reported worldwide with an estimated mortality of 14.3 deaths per 100,000 population with the majority of cases being reported in sub-Saharan Africa and Southeast Asia [5].

The pathophysiology of malaria is based on three key factors, namely (i) red blood cell (RBC) invasion resulting in increased membrane stiffness, thus contributing to luminal sequestration, (ii) upregulation of cell adhesion receptors secondary to increased expression of ring surface protein and *P. falciparum* erythrocyte membrane protein 1 leading to further RBC sequestration, and (iii) malarial toxin (glycophosphoinositol) release which leads to monocyte activation which in turn upregulate inflammatory mediators. The resultant reduction in tissue perfusion, inflammation, and change in interstitial oedema lead to ischaemia and overall organ dysnfuction [6, 7].

The propensity for malaria to cause microvascular damage and obstruction explains why end-organ damage is a hallmark feature of this disease. Postmortem immunohistochemical analysis of retinal tissue obtained from patients who exhibited malarial retinopathy (MR) revealed an increased expression of VEGFR1 (vascular endothelial growth factor receptor 1) as well as aquaporin 4 similar to analyses carried out on cerebral tissue [8]. This suggests that the pathophysiological mechanism underlying MR is in line with the one underlying cerebral malaria [9].

MR is characterized by several key clinical features indicative of microvascular ischaemia, namely (i) patchy retinal whitening in the perimacular area and periphery, (ii) “tram-lining” of the larger calibre vessels and whitening of capillaries and postcapillary venules secondary to thinning of the blood column, (iii) cotton-wool spots, and (iv) retinal haemorrhages with a white core similar to the Roth spots in appearance [10]. In some patients, the presence of papilloedema may also be noted and may be secondary to raised intracranial pressure caused by neurovascular disease, but its presence has been associated with an increased mortality rate if detected in patients suffering from cerebral malaria [11].

The features of MR on OCT are still being studied mainly because of a lack of imaging equipment in remote areas where the disease is more prevalent. A systematic review on the use of retinal imaging in patients suffering from cerebral malaria with concomitant retinopathy carried out by Wilson et al. reported the presence 3 specific OCT biomarkers, namely (i) hyperreflective foci in the inner retina thought to represent capillary cross-sections containing sequestered RBCs, (ii) retinal nerve fibre layer hyperreflectivity in keeping with axonal infarcts, and (iii) hyperreflective bands, sparing the foveola, mostly found in the inner layers of the neural retina [12]. The latter finding was thought to be similar to paracentral acute middle maculopathy based on the location and appearance of the hyperreflective bands on OCT, the pathophysiological process behind MR [13], and the psychophysiological deficit associated with such lesions [14].

AMN was originally described by Bos and Deutman back in 1975 and described as a rare type of outer zonal retinopathy affecting young women [15]. Common symptoms associated with AMN include metamorphopsia, decreased visual acuity, and relative central and/or paracentral scotomata as experienced by the patient described in this report. A study carried out by Sarraf et al. in 2012 showed that AMN represented an ischaemic phenomenon and can be categorized in two distinct types based on the site of ischaemia in relation to the OPL [16]. With the advent of OCT-A, more insight was gained into the pathophysiology behind the two entities with hypotheses suggesting that the two diseases may be related in view of similar microvascular changes detected on imaging [1, 17]. Type I AMN is characterized by hyperreflectivity in the inner nuclear layer (INL) on OCT whilst type II AMN, as depicted in this case report, is associated with hyperreflectivity in the ONL with the INL-OPL junction being the anatomical cut-off, suggesting that the deep capillary plexus could be the main target of the ischaemic event [1, 17]. The absence of capillary cross-sections at the INL/OPL junction and the presence of a draining venule crossing the INL appear to be more indicative of a type II lesion [1].

OCT-A features of both types of AMN have already been described in the literature with deep capillary plexus hypoperfusion being identified as the common mechanism behind both types of ischaemic phenomena [1, 17]. Deep vascular complex flow voids on OCT-A represent deep capillary plexus ischaemia although this feature may not be present in all type II AMN patients [1].

In 2023, Bradly et al. described the presence of both types of AMN in a patient suffering from malaria [3] but, to our knowledge, this is the first case of standalone type II AMN being reported in the literature. It is difficult to establish why and when type I and type II AMN can copresent in a particular case as opposed to exclusively involving a particular layer in such circumstances especially when seeing that both entities are thought to share a common pathophysiological mechanism. To date, the presence of AMN in patients suffering from MR and cerebral malaria has not been given any prognostic bearing due to the lack of studies correlating OCT and OCT-A biomarkers to the systemic effects of malaria [12]. A possible theory could be that since the anatomical site of the deep capillary plexus is, in essence, a water-shed area, then early ischaemic changes may present exclusively below the OPL, thus causing standalone type II AMN. Involvement of the inner layers (supplied by the superficial and intermediate capillary plexi) may, in turn, denote a more profound ischaemic insult which may be mirrored by more severe cerebral ischaemia and a worse systemic prognosis.

In conclusion, this case report provides further evidence supporting the ischaemic effect of malaria on retinal tissue and its presentation on OCT and sheds more light onto the utility of OCT-A when assessing patients suffering from cerebral malaria and MR. Further studies on the clinicopathological differences of the two AMN subtypes and the prognostic significance of imaging biomarkers in patients suffering from malaria will provide more insight into the management of the disease.

## Data Availability

The data used to support the findings in this case report are included within the article.
